# The Hidden World within Plants 2.0

**DOI:** 10.3390/microorganisms11122903

**Published:** 2023-12-01

**Authors:** Essaid Ait Barka, Philippe Jeandet, Rachid Lahlali

**Affiliations:** 1 Unité de Recherche Résistance Induite et Bio-Protection des Plantes, USC INRAe 1488, Université de Reims Champagne-Ardenne, 51100 Reims, France; philippe.jeandet@univ-reims.fr; 2 Phytopathology Unit, Department of Plant Protection, Ecole Nationale d’Agriculture de Meknès, Km10, Rte Haj Kaddour, BP S/40, Menkes 50001, Morocco

Interactions between plants and microorganisms are complex, with some microorganisms causing damage by employing strategies that hinder plant growth and reproduction, while others positively influence plant growth through various physiological activities. This issue focuses on exploring the intricate relationships between plants and microorganisms, which could interact with plant cells and tissues at varying levels of dependence, from mutualism to pathogenicity.

Exploring the hidden world within plants takes center stage in the collection of papers in this issue, each contributing to a deeper understanding of the intricate relationships between microorganisms and their plant hosts. One study investigates the complexity of grapevine trunk diseases, unraveling the potential of endophytic and rhizospheric bacteria as biocontrol agents. Another underlines the light-dependent functions of *Azospirillum brasilense*, a microbial inoculant crucial for agriculture. The broader spectrum of plant–microbe interactions is also addressed, asking fundamental questions about why certain microbes target specific plants and how beneficial microorganisms influence plant immunity. Moreover, this Special Issue highlights the critical role of microorganisms in mitigating the effects of salt stress on lettuce, offering insights into the combined effects of natural bio-stimulants. As a whole, this collection of articles not only provides a comprehensive overview of recent developments in the field but also underscores the importance of the hidden microbial world within plants, emphasizing the need for continued research to uncover more about the complex relationships between plants and microorganisms and their potential applications in sustainable agriculture.

The primary method traditionally used by farmers to mitigate losses due to plant pathogens is the application of fungicides. However, these compounds can become less effective over time, especially under favorable conditions. Further, they pose environmental and human safety concerns. In response to these challenges, a shift toward biological control methods has taken place over the last two decades. Biological control is viewed as a promising, environmentally friendly, and sustainable alternative for the management of plant diseases [1]. This approach involves the use of biological control agents (BCAs) to reduce the amount of pathogen inoculum or inhibit pathogen activity. BCAs employ various mechanisms, including antibiosis, direct parasitism, competition for nutrients and space, and potentially induced resistance [2]. Godana et al. [3] emphasize the specificity of BCAs to their target pathogens, highlighting that they are considered harmless to non-target species. In addition, bacteria in particular, are mentioned as a component of biological control strategies against fungal diseases, contributing to sustainable and environmentally friendly agricultural production [3].

In their study, Legrifi et al. [4] focused on evaluating the potential of ten bacterial strains, isolated from the rhizosphere of citrus and blossoms of pear, apple, and quince trees, for their ability to suppress the mycelial growth of *Pythium schmitthenneri*, a pathogenic agent causing root rot disease in olive trees. These bacterial strains belonging to the genera *Alcaligenes*, *Bacillus*, *Pantoea*, *Sphingobacterium*, and *Stenotrophomonas*, were selected for their antagonistic properties against various pathogens [5]. The researchers conducted *in vitro* assays and found that the bacterial strains exhibited a high efficacy in inhibiting the mycelial growth of *P. schmitthenneri*, with a reduction in mycelial growth exceeding 80%. These antifungal effects were attributed to volatile organic compounds (VOCs) produced by the bacteria, showing inhibition rates ranging from 28.37% to 70.32%. Similar results were also observed by Rani et al. [6]. Additionally, the bacterial cell-free filtrates also demonstrated a significant inhibition of the pathogen mycelial growth. To validate their findings obtained in vitro, Legrifi et al. [4] conducted greenhouse tests. Among the bacterial isolates, *Alcaligenes faecalis* ACBC1 and *Bacillus amyloliquefaciens* SF14 emerged as the most successful in controlling the severity of olive root rot disease. The effectiveness of these strains was linked to their ability to produce lytic enzymes and lipopeptides. The study concluded that these two bacterial isolates, namely ACBC1 and SF14, hold promising potential as biocontrol agents to manage olive root rot disease. The results also suggested that antagonistic bacteria could offer sustainable alternatives to chemical fungicides for controlling this disease.

Grapevine trunk diseases (GTDs), including Botryosphaeria dieback, Eutypa dieback, Phomopsis dieback, esca, and black foot, are caused by various fungal pathogens, leading to significant damage in vineyards [7]. The incidence of GTDs has globally increased over the last three decades. Since the grapevine microbiome has been recognized as a valuable source of biocontrol agents, the concept of a “balanced microbiome” has gained more attention, with research indicating that grapevines with a higher abundance of beneficial endophytic bacteria display fewer symptoms in vineyards with a history of GTDs [8]. The endophytic nature of latent infections caused by trunk pathogens constitutes an advantage for biocontrol treatments, allowing grapevines to strengthen their tolerance to stress [9,10]. However, there is a limited variety of BCAs available to enable growers and nursery managers to effectively control GTDs. To fill this gap, Bustamante et al. [11] conducted a study in California, sampling twenty commercial vineyards to isolate endophytic and rhizospheric bacteria from grapevines with and without GTD symptoms. The researchers identified 1344 bacterial isolates, of which 172 exhibited levels of mycelial growth inhibition over 40% when challenged against *Neofusicoccum parvum* and *Diplodia seriata*. The bacterial isolates responsible for this restriction were mainly identified as *Bacillus velezensis*, *Pseudomonas* spp., and *Serratia plymuthica*. Further testing revealed consistent levels of mycelial inhibition across these bacterial species, with *B. velezensis* demonstrating strong inhibition against *N. parvum* and *Eutypa lata*.

Although traditionally classified as non-phototrophic, *Azospirillum brasilense* possesses photoreceptors that allow it to perceive light, and previous studies have suggested its ability to respond to light stimuli [12]. The study by Housh et al. [13] explores the light dependencies in the biological functions of *A. brasilense*, a bacterium commonly used as a microbial inoculant in agriculture. The study investigated the light dependency of atmospheric biological nitrogen fixation (BNF) and auxin biosynthesis, along with related processes such as ATP biosynthesis and iron and manganese uptake. Functional mutants of *A. brasilense* (HM053, ipdC, and FP10) were studied in both light and dark environments. The results indicated that nitrogenase activity, which is crucial for BNF, exhibited the highest light dependency in the HM053 mutant, followed by the ipdC mutant, while the FP10 mutant did not show significant light dependency. Also, auxin biosynthesis demonstrated strong light dependencies in strains HM053 and FP10 but not in the ipdC mutant. A light dependency was observed in the uptake of ferrous iron (involved in BNF) for HM053 and ipdC mutants but not for FP10. Surprisingly, a light dependency for manganese uptake was only observed in the ipdC mutant. Additionally, ATP biosynthesis was found to be light-sensitive in all three mutants, with a preference for blue light over red light compared to darkness. In conclusion, this study suggested that *A. brasilense*, although non-phototrophic, exhibits light-dependent responses in various biological functions, including nitrogen fixation, auxin biosynthesis, and nutrient uptake [14]. These findings contribute to a better understanding of how environmental factors, such as light, may influence the effectiveness of microbial inoculants in promoting plant growth in agriculture. 

Endophytic plant growth-promoting bacteria, residing asymptomatically in vital plant organs, can be acquired from seeds (vertically) or soils (horizontally) [15,16]. Endophytic seed bacteria, especially in rice, play a crucial role in germination, seedling resistance to climate change, nutrient mobilization, phytopathogen control, antioxidant activity, and hormone production [17]. In their investigation, Hernández et al. [18] focused on the Plant Growth Promoting Rhizobacteria (PGPR) community associated with the rhizosphere of Cuban rice cultivars INCA LP-5 and INCA LP-7. While rhizospheric bacteria have been studied, seed-associated bacteria in these cultivars were unknown. The research aimed to isolate endophytic bacteria from rice seeds and assess their potential to promote plant growth. Nineteen bacterial strains from the genera *Pantoea*, *Bacillus*, *Paenibacillus*, and *Pseudomonas* were isolated from rice seed husks and endosperm. Among these, *Pantoea* sp. S5-1, *Pseudomonas* sp. S5-38, and *Pseudomonas* sp. S7-1 exhibited significant plant growth-promoting traits, including auxin production, phosphate, and potassium solubilization, siderophore production, and inhibition of the phytopathogen *Pyricularia oryzae*. In hydroponic assays, inoculation of these strains improved various growth parameters of rice plants. Similar observations were reported by Redondo-Gómez et al. [19]. The study, conducted by Hernández et al. [18], represents the first report of the presence of endophytic bacteria in the seeds of Cuban rice cultivars. *Pantoea* sp. S5-1, *Pseudomonas* sp. S5-38, and *Pseudomonas* sp. S7-1 are identified as promising strains for the development of bio-stimulants or bio-inoculants intended to improve the growth of Cuban rice crops. This research provided valuable insights on the diversity of bacterial endophytes associated with rice seeds, providing potential applications for sustainable and effective agricultural practices in Cuba.

The study by Ouhaddou et al. [20] explored strategies to mitigate the effects of salt stress on lettuce by employing natural bio-stimulants, including arbuscular mycorrhizal fungi (AMF), PGPR, and compost. The role of PGPRs in inducing physiological, growth, and biochemical changes in plants to cope with extreme conditions has been discussed. Additionally, the application of AMF and organic supplements, such as compost, is presented as promising approaches to confer resilience to abiotic stresses, particularly salinity. Therefore, in their investigation, Ouhaddou et al. [20] evaluated lettuce growth, physiology, enzymatic activities, and soil characteristics in response to these different applications. Consistent with previous reports [21,22], the results indicated that the applied bio-stimulants, especially the combination of AMFs and PGPRs, significantly enhanced the salt tolerance of lettuce, leading to improved growth, physiological traits, and nutrient content under salinity stress. Furthermore, the study suggested that this approach could be effective in rehabilitating saline lands, thus providing a sustainable mitigation option in the context of climate change. The conclusion of this work emphasized that the use of AMFs and PGPRs is a powerful combination for enhancing lettuce tolerance to salt stress. Ouhaddou et al. [20] encourage the use of these bioinoculants in combination with appropriate organic fertilizers in saline land rehabilitation programs for sustainable agricultural systems in the face of climate change.

In conclusion, these studies collectively revealed the hidden world of microorganisms and their diverse roles in agricultural contexts. Legrifi et al. [4] and Bustamante et al. [11] provided valuable insights into biocontrol strategies against olive root rot and grapevine trunk diseases, showcasing the potential of specific bacterial strains as environmentally friendly alternatives to chemical fungicides. Housh et al. [13] revealed unexpected light-dependent responses in *Azospirillum brasilense*, challenging conventional classifications and expanding our understanding of microbial behavior. Hernández et al. [18] uncovered the hidden diversity of endophytic bacteria in Cuban rice seeds, proposing promising candidates for bio-stimulants aimed at boosting crop growth. Lastly, Ouhaddou et al. [20] have explored natural bio-stimulants to alleviate salt stress in lettuce, underscoring the importance of sustainable agricultural practices. Overall, these studies shed light on future research directions, emphasizing the importance of harnessing the microbial potential for resilient and environmentally friendly agricultural systems.
